# Rehydration Driven Na-Activation of Bentonite—Evolution of the Clay Structure and Composition

**DOI:** 10.3390/ma14247622

**Published:** 2021-12-10

**Authors:** Krzysztof Bahranowski, Agnieszka Klimek, Adam Gaweł, Ewa M. Serwicka

**Affiliations:** 1 Faculty of Geology, Geophysics and Environmental Protection, AGH University of Science and Technology, al. Mickiewicza 30, 30-059 Krakow, Poland; aklimek@agh.edu.pl (A.K.); agawel@agh.edu.pl (A.G.); 2 Jerzy Haber Institute of Catalysis and Surface Chemistry, Polish Academy of Sciences, Niezapominajek 8, 30-239 Krakow, Poland; ncserwic@cyf-kr.edu.pl

**Keywords:** bentonite, thermal activation, Na-activation, dry impregnation

## Abstract

A new method of Na-activation of raw bentonite, rich in Ca-montmorillonite, consisting of combined thermal treatment at 200 °C, followed by immediate impregnation with aqueous solution of Na_2_CO_3_ of concentration corresponding to 0.5, 1.0, 1.5, or 2.0 cation exchange capacity (CEC) of clay, was investigated. Structural and compositional evolution of the activated solids after 1, 2, 3, and 4 weeks of storage was monitored by X-ray diffraction (XRD) and Fourier transform infrared spectroscopy (FTIR). XRD analysis indicated that within the investigated period of ageing transformation to Na-rich montmorillonite required Na_2_CO_3_ concentration of at least 1.0 CEC. FTIR spectra showed that, depending on the Na_2_CO_3_ concentration and ageing time, formation of Na-rich montmorillonite was accompanied by precipitation of poorly crystalline calcite, amorphous calcium carbonate, gaylussite (a double calcium-sodium carbonate), and portlandite (Ca(OH)_2_).

## 1. Introduction

Bentonites occupy a special position among clay raw materials due to the unique properties of smectites, their main mineral component. Most common smectites found in bentonites are calcium and/or sodium montmorillonites. Montmorillonite is a phyllosilicate built of layers in which an octahedral Al-based sheet is sandwiched between two tetrahedral Si-based sheets. Partial substitution of Al^3+^_oct_ with Mg^2+^ generates a negative layer charge, compensated by Ca^2+^ and/or Na^+^ cations located in the interlayer. In addition, the interlayer accommodates water molecules. The layer charge of 0.2–0.6 e per half unit cell determines the cation exchange capacity, swellability, and viscosity of bentonite [1], the features of key importance for its use in manufacturing of adsorbents, binders, drilling muds, catalysts, materials for civil engineering, etc. [2]. Smectites are very susceptible to various modifications, which enables tailoring their properties to the requirements of the targeted application. Most frequently, alteration of smectites structure and physico-chemical properties is carried out by means of chemical, thermal or combined chemical-thermal activation [3,4]. Sodium activation, which is an example of chemical modification, is a process developed in the 1930s [5,6]. The discovery was prompted by the observation that only bentonites rich in Na-smectites were suitable for the use as binders in foundry moulding sands, while bentonites containing Ca-smectites proved inadequate for this purpose [4]. Indeed, sodium and calcium montmorillonites display significantly different properties. Ca^2+^ is characterized by higher ionic potential (the ratio of charge to ionic radius), hence its binding in the interlayer is stronger, and hydration coat thicker than in the case of Na^+^. In consequence, Na-rich bentonites are characterized by higher cation exchange capacity, higher swelling properties and better dispersiveness in water than Ca-forms. The patent from 1935, based on invention by German scientists Hofmann and Endell, described the method of transforming Ca-rich bentonites into Na-forms by treatment with sodium carbonate [4,5]. The sodium activation involved addition of 2–2.5 wt.% Na_2_CO_3_ to Ca-rich bentonite in diluted aqueous slurry, followed by washing and drying. Later the process was modified to avoid the costly and time-consuming drying step. To this end, dry Na_2_CO_3_ was added to bentonite rich in Ca-smectite wetted with water in the amount corresponding to 20–30 wt.% of the raw clay, intensely mixed and ground to obtain the Na-activated form [6]. Nowadays soda activation of bentonite rich in Ca-smectite is a standard industrial practice in manufacturing of drilling muds, binders, cat litter, adsorbents, barrier clays, etc. Transformation to Na-form is also commonly employed in multi-stage modifications used in engineering of advanced clay-based materials, as the first step facilitating subsequent phases of the process [7,8]. Due to its importance and widespread use, Na-activation of bentonites has attracted the interest of many researchers and a number of papers addressing this issue have been published [9,10,11,12,13,14,15,16,17,18,19,20,21]. Activation procedures described in the literature involve preparation of mixtures of bentonite, sodium carbonate, and water in various proportions, at different temperatures, interacting for different periods of time, sometimes washed free of excess Na_2_CO_3_, dried or left as suspensions, depending on the purpose of the study (Table 1).

In the present study a novel approach to Na-activation of bentonite is proposed, based on the known effect of spontaneous rehydration and swelling of a dehydrated montmorillonite upon contact with moisture [22,23,24]. The concept involves carrying out rehydration of a dehydrated montmorillonite component of the bentonite with an aqueous solution of Na_2_CO_3_, acting simultaneously as the medium capable of montmorillonite rehydration and the carrier of Na^+^ cations for the exchange process. The method requires preliminary drying of raw Ca-rich bentonite by means of thermal treatment at temperature enabling removal of interlayer water from montmorillonite. Subsequent impregnation with the aqueous solution of Na_2_CO_3_ aims both at the rehydration-driven expansion of clay layers, and the Na^+^ for Ca^2+^ substitution. The impregnation process is designed in such a way that the activated clay remains in a macroscopically dry state, ready for the potential application.

## 2. Materials and Methods

The clay material used in this study was the commercial bentonite from Kopernica deposit in Slovakia (supplied by CERTECH, Niedomice, Poland) containing ca. 80% of Ca-montmorillonite, whose detailed petrographic and mineralogical characterization was published recently (Kopernica 3) [25]. The as received material was homogenized by blending in a ceramic mortar. For Na_2_CO_3_ activation a 100 g sample of bentonite was oven dried (BMT Medical Technology, Brno, Czech Republic) for 3 h at 200 °C to eliminate the interlayer water from montmorillonite. Immediately after removing from the oven, the dried sample was subjected to impregnation with aqueous Na_2_CO_3_ (≥99.5%, Chempur, Piekary Śląskie, Poland) solution and the system homogenized by vigorous stirring. The amount of water used in the impregnation procedure (35 wt.% of the parent clay) was determined in a set of preliminary trial and error experiments, as the maximum volume leaving the sample in the macroscopically dry state (for the purpose of facilitating handling in potential applications). Four samples were prepared, differing in the content of sodium carbonate in the impregnating solutions, i.e., the amount of Na_2_CO_3_ (≥99.5%, Chempur) dissolved in 35 mL of water corresponded to 0.5, 1.0, 1.5, and 2.0 CEC of bentonite (2.1, 4.2, 6.3, and 8.4 g, respectively). Structural characterization of the samples was carried out straight after activation and following 1, 2, 3, and 4 weeks of storage.

Powder X-ray diffraction (XRD) patterns were obtained with a Rigaku SmartLab diffractometer (Rigaku, Tokyo, Japan) for pressed powder samples, using graphite-monochromatized CuKα radiation, operating voltage = 45 kV, current = 200 mA, 2θ step size = 0.05° and counting time = 1 s/step. The measurements were carried out at relative humidity (RH) equal 50%. Crystal size of calcite precipitate was estimated by analyzing the broadening of (112) reflection with Scherrer equation D_hkl_ = Kλ/βcosθ (D_hkl_—coherently scattering domain in the direction perpendicular to the (hkl) plane; K—shape factor, assumed 0.9, λ—incident ray wavelength; θ—Bragg diffraction angle and β—full width at half maximum of the (hkl) reflection.

Fourier transform infrared (FTIR) absorption spectra in middle infrared were recorded with a Nicolet 6700 spectrometer (Thermo Scientific, Madison, WI, USA), in the 4000–400 cm^−1^ range. The samples were prepared by mixing 2 mg of bentonite powder with 200 mg of KBr (Chempur, Piekary Śląskie, Poland) and pressing the mixture into a pellet at 10 MPa pressure. A total of 64 scans at 2 cm^−1^ resolution were taken for each sample.

The cation exchange capacity (CEC) of the parent bentonite was determined using the BaCl_2_ replacement method [26]. In this method CEC is calculated as the sum of exchangeable cations (Ca, Mg, K, Na, Al, Fe, and Mn). A total of 10 mL of 0.1 M BaCl_2_ (Chempur, Piekary Śląskie, Poland) solution was added to 0.1 g of bentonite and thoroughly mixed for 2 h. The mixture was centrifuged (JW Electronic, Lublin, Poland), the supernatant filtered and subjected to analysis for the content of the above listed cations with atomic absorption spectrometry (AAS) method, using Thermo Scientific 3500 (Thermo Electron Manufacturing, Cambridge, UK) equipment. The CEC of the bentonite, determined in such a way, was equal 80 meq/100 g.

Thermal analysis of parent bentonite was carried out with a Netzsch STA 449 F3 Jupiter apparatus (Netzsch, Selb, Germany), at a heating rate of 10 °C/min, with synthetic air flow of 50 mL/min, using ca. 30 mg sample.

## 3. Results and Discussion

### 3.1. Thermal Activation

The proposed method of sodium activation requires preliminary heating of the bentonite, to remove the interlayer water from the montmorillonite component. The temperature of pretreatment was chosen on the basis of thermal analysis data obtained for the parent bentonite and based on considerations of the literature [27,28,29,30].

**Figure 1:**
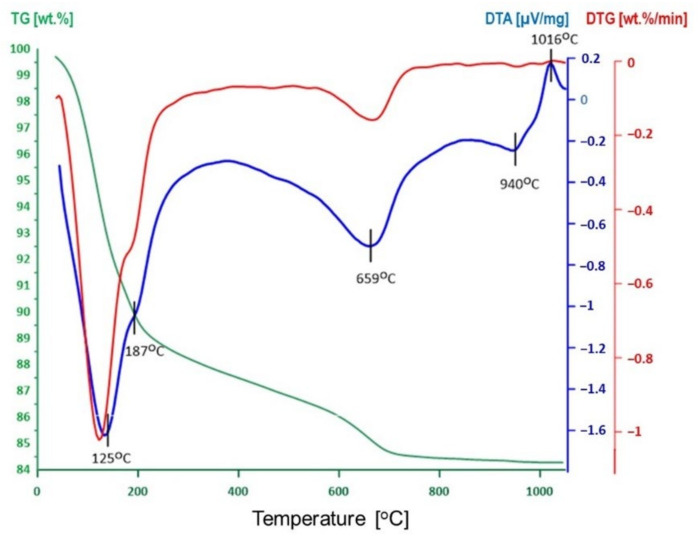
TG/DTG/DTA profiles of parent bentonite.

The TG/DTG/DTA curves of the studied Ca-rich bentonite, shown in Figure 1, are similar to those reported in the literature for Ca-montmorillonites or Ca-rich bentonites [27,28,29,30]. The strong low-temperature endothermic effect with maximum at 125 °C and a shoulder at 187 °C is related to the release of interlayer water. The occurrence of the double peak is consistent with the presence of calcium cations in the interlayer. Due to the high ionic potential of Ca^2+^, the water of coordination leaves the structure at a higher temperature than the remaining interlayer water [27]. The endothermic peaks at 659 and 940 °C are due to the montmorillonite dehydroxylation and decomposition, respectively. The exothermic feature at 1016 °C is related to the formation of new phases. Based on the obtained data and the literature reports indicating 200 °C as the temperature enabling almost complete dehydration of the interlayer in Ca-montmorillonite, without initiating the dehydroxylation process [31,32], this temperature was chosen for the thermal pretreatment stage of Na-activation.

### 3.2. Sodium Activation

**Figure 2:**
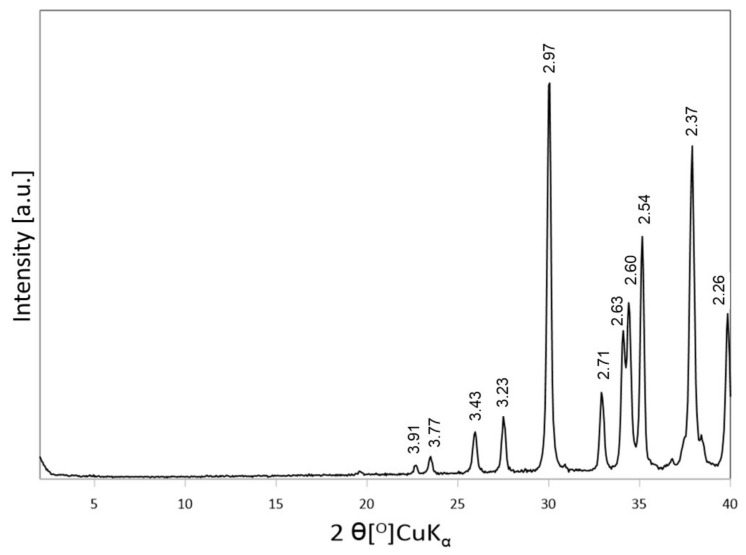
XRD pattern of Na_2_CO_3_ chemical used for the activation.

The XRD pattern of sodium carbonate used for the preparation of impregnating solutions, shown in Figure 2, is characteristic of natrite, an anhydrous form of Na_2_CO_3_ (JCPDS 37-0451), frequently used as an activator in the industrial practice.

#### 3.2.1. XRD Analysis of Activated Bentonites

**Figure 3:**
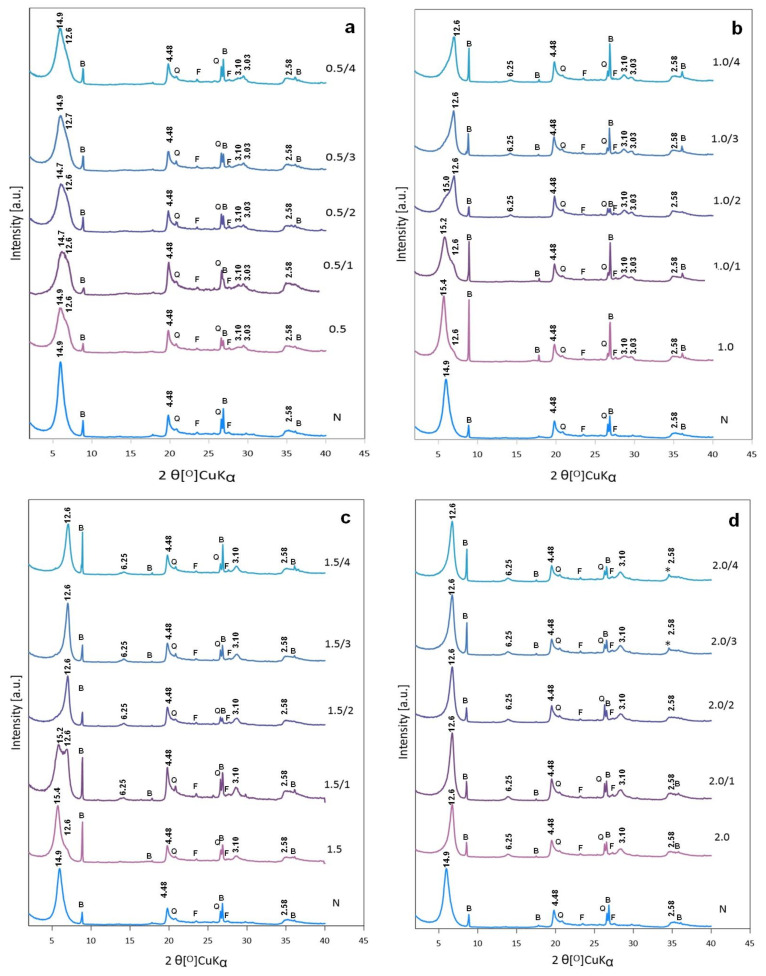
Powder XRD patterns of bentonite activated with Na_2_CO_3_ in quantities corresponding to: (**a**) 0.5 CEC (0.5—freshly activated, 0.5/1—after 1 week, 0.5/2—after 2 weeks, 0.5/3—after 3 weeks, 0.5/4—after 4 weeks) (**b**) 1.0 CEC (1.0—freshly activated, 1.0/1—after 1 week, 1.0/2—after 2 weeks, 1.0/3—after 3 weeks, 1.0/4—after 4 weeks); (**c**) 1.5 CEC (1.5—freshly activated, 1.5/1—after 1 week, 1.5/2—after 2 weeks, 1.5/3—after 3 weeks, 1.5/4—after 4 weeks); (**d**) 2 CEC (2.0—freshly activated, 2.0/1—after 1 week, 2.0/2—after 2 weeks, 2.0/3—after 3 weeks, 2.0/4—after 4 weeks); N—non treated bentonite, B—biotite, Q—quartz, F—feldspar, *—portlandite.

Powder XRD patterns of dried bentonite treated with Na_2_CO_3_ solutions of increasing salt content, to cover 0.5, 1.0, 1.5, and 2.0 CEC of the clay material, are depicted in Figure 3a–d, respectively. The pattern of non-treated bentonite is shown for comparison. The d_001_ value of the basal montmorillonite reflection in the parent clay is 14.9 Å, i.e., consistent with the presence in the interlayer of Ca^2+^ cations embedded in the double layer of water molecules. Mineralogical characterization carried out by Górniak et al. The study [25] identified opal-C/CT, feldspar, biotite, kaolinite, quartz and zeolites, as minority components of the Kopernica bentonite, in various proportions, depending on the mining area. In the Kopernica bentonite investigated in the present study, only biotite, quartz, and feldspar were found. Activation performed with the solution containing the least amount of Na_2_CO_3_ (0.5 CEC) causes an immediate change in the profile of (001) reflection (Figure 3a), which becomes asymmetric and in addition to the maximum at 14.9 Å, a shoulder at ca. 12.7 Å appears. The latter value is characteristic of smectite with a single layer of water in the interlayer. In the adopted experimental conditions such value points to the formation of Na-rich montmorillonite and confirms that a degree of replacement of Ca^2+^ cations with Na^+^ occurred. Co-existence of Ca-rich and Na-rich montmorillonite is further supported by the evolution of 4th order reflection of Na-montmorillonite (d_004_ ~ 3.10 Å). With the Na content used in this experiment, only partial cation exchange is possible. Initially, it is not likely to be homogeneous throughout the whole volume of smectite crystallites, due to the diffusional limitations. The near-to-surface exchange is swifter than penetration of Na^+^ cations into the depth of montmorillonite particle, and diffusion of Ca^2+^ out of the smectite interior. In consequence, in the initial stages of activation, the interlayer spaces at the edges of crystallites are enriched in sodium, while their core is still populated chiefly by calcium cations. Upon ageing the gradients of Na^+^ and Ca^2+^ decrease and, as a result of the system homogenization, the 12.7 Å shoulder of basal reflection becomes less pronounced. After 4 weeks the maximum at 14.9 Å and the overall line shape of the basal reflection, although still asymmetric, show that, despite partial exchange with sodium, the montmorillonite component has predominantly the character of smectite with a double sheet of water in the interlayer, pointing to the decisive role of the remaining interlayer Ca^2+^ cations.

In the conventional methods of bentonite activation with soda, the calcium ions expelled from the interlayer have been reported to precipitate as CaCO_3_ [4,13,16]. Indeed, in Figure 3a, a weak, broad peak corresponding to d ~ 3.03 Å, attributable to the most intense (112) reflection of poorly crystalline calcite (JCPDS 47-1743), is visible. XRD patterns of bentonite samples treated with Na_2_CO_3_ in the amount corresponding to 1.0 CEC of clay are gathered in Figure 3b. Directly after activation the dominant reflection maximally corresponds to d_001_ ~ 15.4 Å, with a shoulder ~ 12.6 Å, indicating that although some exchange of calcium cations with Na^+^ occurred, the interlayer of montmorillonite is still occupied mainly with Ca^2+^. After one week of ageing the relative intensity of the shoulder corresponding to Na-form increases, and after two weeks the maximum associated with Na-montmorillonite becomes the dominant feature, while the component due to Ca-form loses intensity and appears as a shoulder only. Additional ageing for 3 and 4 weeks leads to further increase of 12.6 Å reflection at the expense of the one around 15 Å, so that the contribution of the latter component is visible only as a peak asymmetry tailing off towards lower 2Ɵ values. The evolution of basal reflection of Na-rich form is accompanied by the appearance of 2nd and 4th order reflections (d_002_ ~ 6.2 Å, d_004_ ~ 3.1 Å), in accordance with the 12.6 Å peak assignment. The gradual transformation of Ca-montmorillonite into Na-rich form proves that cation exchange of Ca^2+^ with Na^+^ continues for some time after the impregnating treatment. Similarly, as in the previously discussed case, a broad, low-intensity peak corresponding to d = 3.03 Å indicates that activation is associated with precipitation of CaCO_3_ in the form of poorly crystalline calcite. The crystal size of calcite in these samples, estimated by means of the Scherrer equation, is in the range 60–100 nm.

The evolution of XRD patterns of bentonite activated with yet more concentrated Na_2_CO_3_ solution (soda amount corresponding to 1.5 CEC of bentonite) is illustrated in Figure 3c. Qualitatively, the effect of the treatment is similar to that observed with Na_2_CO_3_ amount equal 1.0 CEC, but it is evident that upon treatment with more concentrated solution the exchange of Ca^2+^ with Na^+^ is swifter and already after 2 weeks of ageing the XRD pattern shows a single basal reflection with maximum corresponding to d_001_ ~ 12.6 Å. The line shape of (001) reflection after 3 and 4 weeks is symmetric and does not change, indicating that the process of cation exchange neared equilibrium. Interestingly, no trace of the most intense reflection of calcite (d = 3.03 Å) can be found in any of the diffractograms. The absence of calcite appears surprising, bearing in mind that it can be found in XRD patterns of bentonites activated with lower content of soda, in which the amount of exchanged calcium ions cannot be complete (Figure 3a,b). Moreover, at the content of Na_2_CO_3_ corresponding to 1.5 CEC of clay, the activation product is expected to contain the excess of soda. However, no crystalline forms of Na_2_CO_3_ can be detected in the XRD patterns of activated samples.

In the case of the highest employed Na_2_CO_3_ amount (2.0 CEC of parent bentonite) the cation exchange is so quick that already the material analyzed directly after exchange shows only the basal reflection typical of the Na-form of montmorillonite, and the XRD pattern does not change in a meaningful way upon ageing. Also, in this series of experiments, no trace of calcium carbonate can be detected in the activated solid. Similarly, no evidence of crystallization of excess Na_2_CO_3_ can be found. Noteworthy, in the samples aged for 3 and 4 weeks, a small reflection, marked with an asterisk, appears. Its position corresponds to d ~ 2.6 Å, i.e., matches with the most intense reflection of portlandite, Ca(OH)_2_, (JCPDS 44-1481), known to form in cementitious materials in strongly basic conditions [33]. Apparently, in the clay activated with the highest concentration of Na_2_CO_3_, formation of traces of portlandite from Ca cations pushed out of the interlayer is feasible.

#### 3.2.2. FTIR Analysis of Activated Bentonites

**Figure 4:**
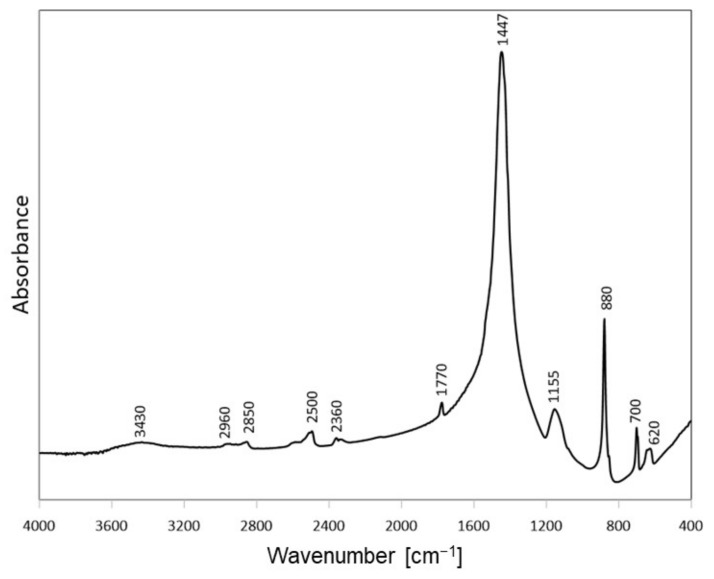
FTIR spectrum of Na_2_CO_3_ chemical used for the activation.

FTIR spectrum of sodium carbonate used in the activation procedure (Figure 4) is dominated by the band at 1447 cm^−1^, characteristic of ν_3_ asymmetric stretching vibrations of C-O bonds in carbonate anion, and resembles closely the previously reported spectra of natrite samples from different manufacturers [16].

**Figure 5:**
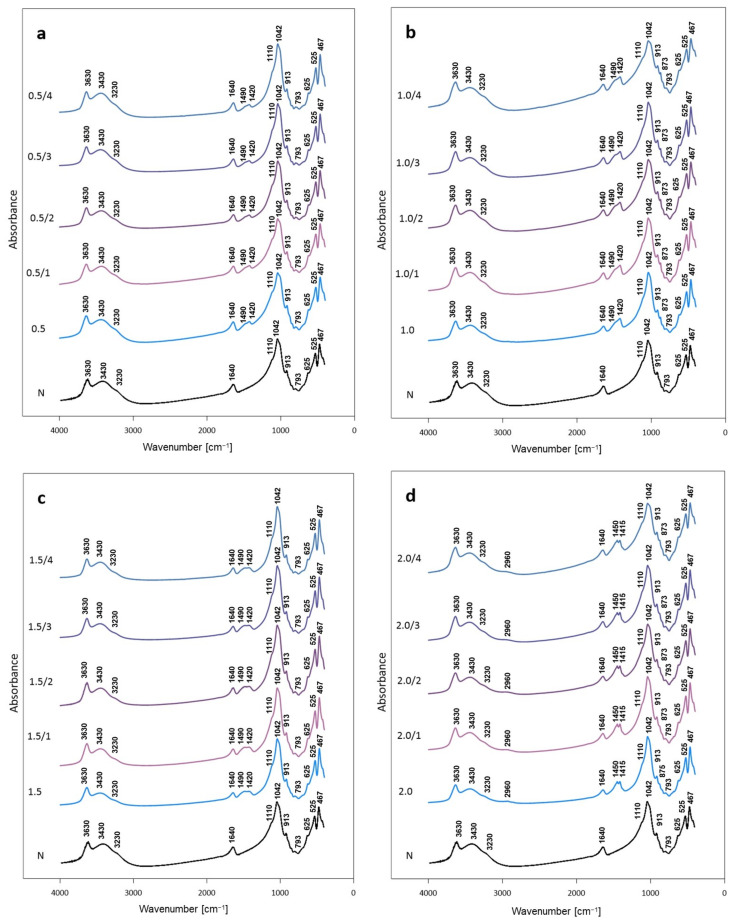
FTIR patterns of bentonite activated with Na_2_CO_3_ in quantities corresponding to: (**a**) 0.5 CEC (0.5—freshly activated, 0.5/1—after 1 week, 0.5/2—after 2 weeks, 0.5/3—after 3 weeks, 0.5/4—after 4 weeks) (**b**) 1.0 CEC (1.0—freshly activated, 1.0/1—after 1 week, 1.0/2—after 2 weeks, 1.0/3—after 3 weeks, 1.0/4—after 4 weeks); (**c**) 1.5 CEC (1.5—freshly activated, 1.5/1—after 1 week, 1.5/2—after 2 weeks, 1.5/3—after 3 weeks, 1.5/4—after 4 weeks); (**d**) 2 CEC (2.0—freshly activated, 2.0/1—after 1 week, 2.0/2—after 2 weeks, 2.0/3—after 3 weeks, 2.0/4—after 4 weeks); N non treated bentonite.

Figure 5a–d shows the FTIR spectra of bentonite treated with Na_2_CO_3_ solutions of increasing salt content, directly after activation and after ageing for 1, 2, 3 and 4 weeks. The spectrum of untreated bentonite is typical of montmorillonite-rich raw clay material [34,35]. In the OH stretching region the band at 3630 cm^−1^ stems from vibrations of OH groups coordinated to different octahedral cations, the broad band around 3430 cm^−1^ is due to stretching vibrations of OH-groups in hydrogen bonded water molecules, while the shoulder at 3230 cm^−1^ is attributed to the overtone of H_2_O bending vibration responsible for the broad band at 1640 cm^−1^. The band with maximum at 1042 cm^−1^ is due to in-plane Si–O–Si stretches and the shoulder around 1110 cm^−1^ originates from perpendicular Si–O vibrations. The 913 cm^–1^ band stems from the Al–Al–OH bending mode, the 793 cm^–1^ band indicates the presence of quartz, the 625 cm^–1^ band stems from out-of-plane Al–O–Si vibrations, and the bands at 525 cm^–1^ and 467 cm^–1^ are related to Al_oct_–O–Si, and Si–O–Si bending vibrations, respectively.

In FTIR spectra of soda-activated bentonite, next to the features characteristic of montmorillonite, new bands appear. In materials activated with soda solutions covering 0.5 and 1.0 CEC of bentonite, a broad band with maximum at 1420 cm^−1^ and a shoulder around 1490 cm^−1^ is observed in the range where ν_3_ asymmetric stretching vibrations of carbonate are expected (Figure 5a,b). The band at 1420 cm^−1^ is due to the ν_3_ mode of carbonate group in calcite [36], whose formation in the investigated samples was evidenced by XRD analysis. Formation of calcite is also responsible for the appearance of the 873 cm^−1^ band due to the out-of-plane deformation mode of carbonate anion. The presence of shoulder at 1490 cm^−1^ indicates that apart from XRD-detectable, poorly crystalline calcite, also amorphous calcium carbonate is formed. Due to the lowering of symmetry of carbonate anion in amorphous solid, the ν_3_ mode becomes split and appears as 1490 and 1425 cm^−1^ doublet with comparable intensities of both components [36]. In the present case, the 1425 cm^−1^ absorption overlaps with the 1420 cm^−1^ mode of calcite, enhancing the band intensity, and the 1490 cm^−1^ mode forms a shoulder of lower intensity.

Upon treatment of bentonite with the amount of Na_2_CO_3_ corresponding to 1.5 CEC, a change in the appearance of the carbonate bands in the 1400–1500 cm^−1^ range occurs (Figure 5c). The 1490 and 1425 cm^−1^ absorptions of similar intensities point to the presence of amorphous CaCO_3_ [36]. In agreement with the XRD result, there is no indication of the formation of calcite. Amorphous CaCO_3_ may form as a precursor to a crystalline state, but usually is short-lived and readily transforms to crystalline modifications. However, it has been observed that crystallization of amorphous precipitate may be hindered by increasing the pH of the environment [37]. In view of this, the increased basicity of a solution with a higher content of Na_2_CO_3_ is the most likely reason for stabilization of CaCO_3_ in an amorphous form. From the FTIR spectra it is not possible to conclude unequivocally on the presence of the excess soda, because the ca. 1450 cm^−1^ carbonate band of Na_2_CO_3_ [38], would overlap with the doublet characteristic of amorphous CaCO_3_.

In the bentonite activated with the most concentrated soda solution, covering 2.0 CEC of clay, two well-resolved maxima at 1450 cm^−1^ and 1415 cm^−1^ become visible in the area of ν_3_ carbonate modes. In addition, a broad, low-intensity band around 2960 cm^−1^ appears. In view of the fact that in this sample half of introduced Na_2_CO_3_ constitutes the excess with respect to CEC, the 1450 cm^−1^ band may be attributed to carbonate vibration in the surplus soda. On the other hand, the appearance of the 1415 cm^−1^ maximum, along with the feature around 2960 cm^−1^, points to the formation of a carbonate with sodium and calcium in the structure, known as gaylussite, Na_2_Ca(CO_3_)_2_∙5H_2_O [38,39,40]. The 2960 cm^−1^ band is due to stretching mode of water of crystallization in this compound [39]. The small band at 875 cm^−1^ may be attributed to the out-of-plane deformation mode of carbonate in gaylussite. Gaylussite may form as a result of reaction between CaCO_3_ and sufficiently concentrated Na_2_CO_3_ solution [41]. Apparently, this condition is fulfilled at the highest Na_2_CO_3_ content used in this study (ca. 8 wt.% of the parent clay).

With the soda amount corresponding to 2.0 CEC of clay, half of the introduced sodium should be consumed in the cation exchange, assuming that the process is complete. The other half, which remains outside the clay particles, and the calcium cations expelled from the interlayer, are in 2:1 molar proportion, which corresponds to the stoichiometry of gaylussite. The FTIR-detected presence of the unreacted Na_2_CO_3_, next to gaylussite, implies that some other calcium-containing compounds must also be present. Indeed, the trace of portlandite has been detected by XRD. FTIR cannot corroborate the presence of Ca(OH)_2_, because its strongest mode, associated OH stretches, overlaps with the 3630 cm^−1^ band of montmorillonite [42]. Amorphous CaCO_3_ is another possible component of the activated clay. However, even if present, its broad bands are not resolved, and remain hidden in the complex line shape of the asymmetric stretching modes of other carbonates.

#### 3.2.3. Influence of Na_2_CO_3_ Concentration

The specific feature of the soda activation procedure proposed in this work is that the aqueous soda solution, when poured over bentonite dried at 200 °C, acts, on one hand, as a medium enabling spontaneous rehydration of the montmorillonite interlayer, and, on the other, as a source of sodium cations for exchange reaction with calcium cations. Moreover, it may act as a reagent interacting with the chemical compounds available in the system.

XRD analysis of samples directly after activation and during 4 weeks of ageing shows that the concentration of the impregnating solution represents an efficient tool in controlling the progress of activation. The Na^+^ for Ca^2+^ exchange is driven by the difference of cation concentrations between the interlayer and the solution. While the initial gradient of Ca^2+^ concentration is constant, determined by the Ca^2+^ content in the interlayer, the initial gradient of Na^+^ may be modified by adjusting the amount of Na_2_CO_3_ in the solution. Upon increasing soda concentration, the endpoint of structural transformations is achieved in ever shorter time. Thus, it takes ca. 4 weeks for equilibration of the system treated with soda solution covering 0.5 CEC, but for the Na_2_CO_3_ amount corresponding to 2.0 CEC an almost instantaneous transformation of Ca-rich bentonite into Na-form is observed.

Besides affecting the rate of structural transformation, the concentration of impregnating solution determines the degree of Na^+^ for Ca^2+^ substitution. To obtain Na-rich bentonite the solution of Na_2_CO_3_ has to cover at least 1.0 CEC of clay.

Combined XRD and FTIR data reveal that concentration of Na_2_CO_3_ influences also the fate of calcium cations driven out of the interlayer. At low soda content (0.5 and 1.0 CEC) Ca^2+^ precipitates as XRD detectable, poorly crystalline calcite. In addition, FTIR spectra show that calcite crystallites coexist with amorphous CaCO_3_. At Na_2_CO_3_ content corresponding to 1.5 CEC, crystallization of CaCO_3_ is hampered by increased basicity of the impregnating solution, and only amorphous calcium carbonate can exist in the system. Further increase of Na_2_CO_3_ concentration (2.0 CEC) creates conditions for the occurrence of reaction between Na_2_CO_3_ solution and CaCO_3_ precipitate, leading to the formation of gaylussite, the sodium-calcium carbonate, identified by FTIR analysis. This strongly basic environment enables also formation of portlandite, whose traces are detected by XRD.

## 4. Conclusions

The new method of soda activation of Ca-rich bentonite, involving dehydration of clay at 200 °C, followed by rehydration with aqueous solution of Na_2_CO_3_, enables easy preparation of Na-rich bentonite, in a manner requiring less processing steps than most of the procedures described in the literature (Table 1). Notably, the volume of liquid is adjusted in such a way as to leave the clay in an apparently dry state, thus eliminating the usually required drying step (Table 1) and facilitating further handling.

XRD and FTIR characterization of structural and compositional evolution of the activated bentonite indicates that the degree of Na for Ca substitution may be controlled by an appropriate choice of the concentration of soda solution and the time of treatment.

Depending on the concentration of Na_2_CO_3_, and the time of ageing, the auxiliary compounds formed during activation from calcium cations released from the montmorillonite lattice encompass poorly crystalline calcite, amorphous CaCO_3_, gaylussite (a double Na-Ca carbonate), and portlandite (Ca(OH)_2_).

## Figures and Tables

**Table 1:** Examples of Na-activation procedures described in the literature (Bent—bentonite, Mt—montmorillonite).

Activation Procedure	Purpose	Reference
Mixture of Bent, water (24 or 34 wt.% of total dried matter) and Na_2_CO_3_ (1.5, 2.2 or 3.5 wt.% of clay) kneaded in a mill for 10 min and oven-dried at 80 °C for 3 h, ball-milled and sieved.	Study of ageing process with respect to rheological properties	[9]
Dry Mt added to saturated solution of Na_2_CO_3_ (100 meq/100 g Mt) homogenized at RT, left standing for 24 h and diluted to 6 wt.% aqueous suspension.	Study of rheological properties	[10,12]
100 g of Ca-Bent and Na_2_CO_3_ (0.1, 2, 2.5, 5, 10 or 15 wt.% of clay) stirred in 800 mL boiling water for 1 h, clay fraction separated by multiple dispersion/sedimentation cycles. Supernatant concentrated by evaporation and the activated clay dried at 105 °C and ground.	Study of rheological properties	[11]
Bent moisturized with 40 wt.% water mixed with dry Na_2_CO_3_, mixture kneaded at RT and left to drying/curing under sunlight for a month. Alternatively, Na_2_CO_3_ blended with MgO.	Study of rheological properties (for application as drilling fluid)	[14]
Mixture of moisturized Bent and Na_2_CO_3_ (2, 3, 5, 10 wt.% of clay) kneaded at RT, left to drying/curing under sunlight for a month, mixed with water (30, 50 and 75 g/L) and the suspension aged for 24 h.	Study of rheological properties (for application as drilling fluid)	[17]
Na_2_CO_3_ (2, 4, 12 wt.% of clay) added to 6 wt.% suspension of Ca-Bent, stirred with or without heating at 70 °C, and aged for 24 h.	Study of rheological properties (for application as drilling fluid)	[19]
22.5 g of Bent stirred in 350 mL of seawater, alkalized with NaOH to pH = 9, mixed with Na_2_CO_3_ (0.5, 1, 1.5 g), left under heating and stirring at 100 °C for 24 h, filtered and dried.	Study of rheological properties (for application as drilling fluid)	[20]
Mixture of 100 g of clay and Na_2_CO_3_ (2, 3, 5, 8 wt.% of clay) stirred in 1000 mL boiling water for 1 h, cooled and purified by multiple dispersion/sedimentation cycles. The activated clay collected by centrifugation, dried at 60 °C and ground.	Pharmaceutical application	[15]
Bent mixed with 2–5% Na_2_CO_3_ and various amounts of water (10–15% or excess), allowed to interact under shaking for 24 h, and dried at 60 °C.	Study of differences between natural and activated Na-Bent	[16]
Commercial Na-activated Bent obtained by treatment of wet raw Bent with solid Na_2_CO_3_ (3 wt.% of clay) at 80 °C.	Study of protein sorption (for application in winemaking)	[13]
Mixture of 5 g of clay and Na_2_CO_3_ (2, 3, 5, 10 wt.% of clay) stirred in 150 mL water for variable activation times (1, 2, 4 h), dried at 60 °C without washing.	Study of adsorptive properties (for application in edible oil clarification)	[18]
Na_2_CO_3_ (4 wt.% of clay) added to a suspension of Bent in 500 mL boiling water, stirred for 1 h, and cooled. The clay recovered by sedimentation, separated by filtering, washed, dried at 105 °C and calcined at 450 °C for 24 h.	Study of adsorptive properties (for waste water purification)	[21]

## Data Availability

The data presented in this study are available on request from corresponding author.

## References

[1] Christidis G.E., Brigatti M.F., Mottana A. (2011). The concept of layer charge of smectites and its implications for important smectite-water properties. Layered Mineral Structures and Their Application in Advanced Technologies.

[2] Murray H.H., Murray H.H. (2006). Bentonite Applications. Applied Clay Mineralogy: Occurrences, Processing and Applications of Kaolins, Bentonites, Palygorskite-Sepiolite, and Common Clays.

[3] Heller-Kalai L., Bergaya F., Lagaly G. (2013). Thermally modified clay minerals. Handbook of Clay Science. Part A: Fundamentals.

[4] Harvey C.C., Lagaly G., Bergaya F., Lagaly G. (2013). Industrial application. Handbook of Clay Science. Part B: Techniques and Applications.

[5] Erbslöh S. (1935). Improved Manufacture of Highly Swellable Inorganic Substances. GB Patent.

[6] Erbslöh S. (1936). Improved Manufacture of Highly Swellable Inorganic Substances. GB Patent.

[7] He H., Frost R.L., Deng F., Zhu J., Wen X., Yuan P. (2004). Conformation of Surfactant Molecules in the Interlayer of Montmorillonite Studied by ^13^C MAS NMR. Clays Clay Miner..

[8] Yuan P., He H.P., Bergaya F., Wu D.Q., Zhou Q., Zhu J.X. (2006). Synthesis and characterization of delaminated iron-pillared clay with meso-microporous structure. Micropor. Mesopor. Mater..

[9] Lebedenko F., Plée D. (1988). Some considerations on the ageing of Na_2_CO_3_-activated bentonites. Appl. Clay Sci..

[10] Volzone C., Garrido L.B. (1991). The effect of some physico-chemical and mineralogical properties on the Na_2_CO_3_ activation of Argentine bentonites. Appl. Clay Sci..

[11] Yildiz N., Sarikaya Y., Çalimli A. (1999). The effect of the electrolyte concentration and pH on the rheological properties of the original and the Na_2_CO_3_-activated Kütahya bentonite. Appl. Clay Sci..

[12] Volzone C., Garrido L.B. (2001). Changes in suspension properties of structural modified montmorillonites. Cerâmica.

[13] Gougeon R.D., Soulard M., Miehé-Brendlé J., Chézeau J.M., Le Dred R., Jeandet P., Marchal R. (2003). Analysis of Two Bentonites of Enological Interest before and after Commercial Activation by Solid Na_2_CO_3_. J. Agric. Food Chem..

[14] Karagüzel C., Çetinel T., Boylu F., Çinku K., Çelik M.S. (2010). Activation of (Na, Ca)-bentonites with soda and MgO and their utilization as drilling mud. Appl. Clay Sci..

[15] Shah L.A., Khattak N.S., Valenzuela M.G.S., Manan A., Valenzuela Díaz F.R. (2013). Preparation and characterization of purified Na-activated bentonite from Karak (Pakistan) for pharmaceutical use. Clay Miner..

[16] Kaufhold S., Emmerich K., Dohrmann R., Steudel A., Ufer K. (2013). Comparison of methods for distinguishing sodium carbonate activated from natural sodium bentonites. Appl. Clay Sci..

[17] Boussen S., Sghaier D., Chaabani F., Jamoussi B., Messaoud S.B., Bennour A. (2015). The rheological, mineralogical and chemical characteristic of the original and the Na_2_CO_3_-activated Tunisian swelling clay (Aleg Formation) and their utilization as drilling mud. Appl. Clay Sci..

[18] Mosbahi M., Tlili A., Khlifi M., Jamoussi F. (2017). Basic activation of lower Eocene clay from Meknassy-Mezzouna basin (centerwestern Tunisia), synthesis of zeolite and clarification of soybean oils. Appl. Clay Sci..

[19] Magzoub M.I., Nasser M.S., Hussein I.A., Benamor A., Onaizi S.A., Sultan A.S., Mahmoud M.A. (2017). Effects of sodium carbonate addition, heat and agitation on swelling and rheological behavior of Ca-bentonite colloidal dispersions. Appl. Clay Sci..

[20] Mahmoud M., Mohamed A., Kamal M.S., Sultan A.S., Hussein I.A. (2019). Upgrading Calcium-Bentonite to Sodium-Bentonite Using Seawater and Soda Ash. Energy Fuels.

[21] El Ouardi Y., Lenoble V., Branger C., Laatikainen K., Angeletti B., Ouammou A. (2021). Enhancing clay adsorption properties: A comparison between chemical and combined chemical/thermal treatments. Groundw. Sustain. Dev..

[22] Kittrick J.A. (1969). Interlayer Forces in Montmorillonite and Vermiculite. Soil Sci. Soc. Am. J..

[23] Sato T., Watanabe T., Otsuka R. (1992). Effects of Layer Charge, Charge Location, and Energy Change on Expansion Properties of Dioctahedral Smectites. Clays Clay Miner..

[24] Laird D.A. (1996). Model for crystalline swelling of 2:1 phyllosilicates. Clays Clay Miner..

[25] Górniak K., Szydłak T., Gaweł A., Klimek A., Tomczyk A., Sulikowski B., Olejniczak Z., Motyka J., Serwicka E.M., Bahranowski K. (2016). Commercial bentonite from the Kopernica deposit (Tertiary, Slovakia): A petrographic and mineralogical approach. Clay Miner..

[26] Hendershot W.H., Lalande H., Duquette M., Carter M.R., Gregorich E.G. (2008). Ion Exchange and Exchangeable Cations. Soil Sampling and Methods of Analysis.

[27] Wacławska I. (1984). Dehydration and dehydroxylation of smectites 1. Dehydration and dehydroxylation kinetics. Mineral. Pol..

[28] Fajnor V.Š., Jesenák K. (1996). Differential thermal analysis of montmorillonite. J. Therm. Anal..

[29] Dellisanti F., Calafato A., Pini G.A., Moro D., Ulian G., Valdrè G. (2018). Effects of dehydration and grinding on the mechanical shear behaviour of Ca-rich montmorillonite. Appl. Clay Sci..

[30] Rouquerol F., Rouquerol J., Llewellyn P., Bergaya F., Lagaly G. (2013). Thermal analysis. Handbook of Clay Science. Part B: Techniques and Applications.

[31] Lang L.Z., Xiang W., Huang W., Cui D.S., Schanz T. (2017). An experimental study on oven-drying methods for laboratory determination of water content of a calcium-rich bentonite. Appl. Clay Sci..

[32] Derkowski A., Drits V.A., McCarty D.K. (2012). Rehydration of dehydrated-dehydroxylated smectite in a low water vapor environment. Am. Miner..

[33] Dathe F., Strelnikova V., Werling N., Emmerich K., Dehn F. (2021). Influence of lime, calcium silicate and portlandite on alkali activation of calcined common clays. Open Ceramics.

[34] Madejová J., Gates W.P., Petit S., Gates W.P., Klopproge J.T., Madejová J., Bergaya F. (2017). IR spectra of clay minerals. Infrared and Raman Spectroscopies of Clay Minerals. Developments in Clay Science.

[35] Farmer V.C., Farmer V.C. (1974). The layer silicates. Infrared Spectra of Minerals.

[36] Andersen F.A., Brečević L. (1991). Infrared spectra of amorphous and crystalline calcium carbonate. Acta Chem. Scand..

[37] Tobler D.J., Rodriguez Blanco J.D., Sørensen H.O., Stipp S.L.S., Dideriksen K. (2016). Effect of pH on Amorphous Calcium Carbonate Structure and Transformation. Cryst. Growth Des..

[38] Estep P.A., Kovach J.J., Hiser A.L., Karr C., Friedel R.A. (1970). Characterization of Carbonate Minerals in Oil Shales and Coals by Infrared Spectroscopy. Spectrometry of Fuels.

[39] Böttcher M.E., Gehlken P.L. (1996). Dehydration of natural gaylussite (Na_2_Ca(CO_3_)_2_∙5H_2_O) and pirssonite (Na_2_Ca(CO_3_)_2_∙2H_2_O) as illustrated by FTIR spectroscopy. Neues Jahrb. Mineral. Mon..

[40] Frost R.L., Dickfos M. (2007). Hydrated double carbonates—A Raman and infrared spectroscopic study. Polyhedron.

[41] Bury C.R., Redd R. (1933). The system sodium carbonate–calcium carbonate–water. J. Chem. Soc..

[42] Horgnies M., Chen J.J., Bouillon C. (2013). Overview about the use of Fourier transform infrared spectroscopy to study cementitious materials. WIT Trans. Eng. Sci..

